# IrriDrone: Overhead drone-based video dataset for precision sprinkler irrigation assessment

**DOI:** 10.1016/j.dib.2026.113208

**Published:** 2026-08-27

**Authors:** Somayeh Afshar, Mohsen Ramezani, Kaveh Mollazade, Atefe Moradiani

**Affiliations:** a Department of Computer Engineering, Faculty of Engineering, University of Kurdistan, Sanandaj, Iran; b Department of Biosystems Engineering, Faculty of Agriculture, University of Kurdistan, Sanandaj, Iran

**Keywords:** Drone-based monitoring, Precision irrigation, Computer vision, Aerial video analysis, Agricultural water management

## Abstract

Efficient water management in agriculture is a growing concern due to increasing water scarcity and over-reliance on groundwater resources. Despite advances in remote sensing and drone-based monitoring, the lack of standardized and publicly available datasets for vision-based sprinkler irrigation analysis has limited progress in the development of data-driven irrigation management systems. In this work, we present IrriDrone, a new dataset comprising aerial videos and corresponding quantitative water-discharge measurements collected from real-world sprinkler irrigation operations in carrot, potato, and alfalfa fields. Three operational pressure conditions were established by simultaneously operating 3, 6, or 9 sprinklers under an unchanged pump setting, representing high, intermediate, and low pressure per sprinkler, respectively; the corresponding discharge rates were determined in liters per second using a repeated volumetric measurement. The dataset captures variations in sprinkler configuration, drone flight altitude (35, 45, and 55 m), and time of day, and includes GPS metadata and paired video versions with and without manually defined circular overlays representing the irrigation spray boundaries. By providing aerial visual data together with corresponding quantitative irrigation measurements, the dataset supports the development and evaluation of machine learning and computer vision methods for irrigation monitoring and precision water management. The dataset includes continuous recordings covering sprinkler rotations, allowing flexible temporal segmentation for diverse downstream research tasks. To the best of our knowledge, IrriDrone is among the first publicly available datasets to combine overhead drone video with corresponding water-discharge measurements under real agricultural conditions.

Specifications Table

**Table:** 

Subject	Computer Sciences
Specific subject area	Drone-based computer vision for precision irrigation assessment and agricultural water management
Type of data	Standardized aerial videos with and without spray-boundary overlays (.MP4), tabular metadata (.CSV), and dataset documentation (.txt).
Data collection	Aerial videos were collected from sprinkler-irrigated carrot, potato, and alfalfa fields using a DJI Air 3S drone under configurations of 3, 6, and 9 simultaneously active sprinklers. Immediately after each recording, water discharge was determined in liters per second using three repeated 20-L volumetric measurements under unchanged irrigation-system settings.
Data source location	Ghoruchay village, Dehgolan County, Kurdistan Province, Iran Latitude: 35°22’ N Longitude: 47°16’ E
Data accessibility	Repository name: Zenodo Data identification number: 10.5281/zenodo.22017449 Direct URL to data: 10.5281/zenodo.22017449 Version: 1.0 License: Creative Commons Attribution 4.0 International (CC BY 4.0)
Related research article	Not applicable

## 1 Value of the Data

• To the best of our knowledge, IrriDrone is among the first publicly available datasets to combine overhead drone videos of sprinkler irrigation with corresponding quantitative water-discharge measurements under real agricultural conditions. This combination enables investigation of the relationship between visible spray patterns and measured water discharge.
• The dataset is relevant to researchers and practitioners in computer vision, precision agriculture, irrigation engineering, agricultural remote sensing, and water-resource management. It can support tasks such as spray-boundary identification, sprinkler coverage assessment, water-discharge estimation, irrigation anomaly detection, and temporal analysis of sprinkler operation.
• Recordings from three crop fields, three active-sprinkler configurations, three drone flight altitudes, and different times of day provide variation in crop canopy, viewing scale, illumination, shadows, background appearance, and visible spray behavior. Associated GPS coordinates, flight altitudes, recording times, and discharge measurements facilitate condition-specific and spatio-temporal analyses.
• For each boundary-overlaid video, a corresponding standardized video without the circular overlay is provided, allowing researchers to inspect the reference spray boundary while retaining access to the unobstructed visual content for downstream analysis. The two versions represent paired forms of the same recording and should not be treated as independent training and test samples.
• The continuous recordings can be divided into shorter temporal segments according to the requirements of different downstream tasks. To reduce temporal leakage, all clips derived from the same continuous source recording should be assigned to the same training, validation, or test subset.

## 2 Background

Efficient irrigation monitoring is increasingly important for improving agricultural water management [1]. Precision irrigation approaches aim to assess water distribution and irrigation-system operation so that water use can be better managed without compromising crop production [1,2]. Unmanned aerial vehicles provide high-resolution and repeatable observations of agricultural fields and have been used to monitor crop condition, soil moisture, water stress, and irrigation distribution [[3], [4], [5]]. The combination of aerial imagery with field measurements can support computer vision and machine learning methods for analyzing irrigation operation and water distribution.

Despite advances in agricultural remote sensing, the availability of datasets specifically designed for vision-based analysis of sprinkler operation remains limited [6,7]. Existing resources commonly focus on crop classification, soil moisture, vegetation indices, evapotranspiration, or plant water stress using static RGB, multispectral, or thermal imagery [[8], [9], [10]]. Few datasets provide continuous overhead video of sprinkler operation together with corresponding quantitative discharge measurements and reference information about the visible spray extent. Table 1 compares representative datasets and studies in agricultural monitoring and irrigation-related research.

**Table 1:** Summary of related datasets in agricultural monitoring and irrigation research.

Dataset / Study	Crop / Field	Data Type	Sensors / Tools	Key Features	Flight / Recording Conditions	Limitation
Albuquerque et al. [2]	Simulated sprinkler irrigation	RGB video frames (∼300 frames)	Custom quadrotor + Nikon D330	700 annotated spray images, Mask R-CNN training	Artificial irrigation setup; ∼300 frames extracted from video	Small-scale and simulated rather than farm-based; no quantitative water-discharge measurements
Cucho-Padin et al. [9]	Potato (cv. UNICA)	Thermal + RGB images + physiological data (leaf temp, stomatal conductance)	FLIR E60 thermal camera	Dataset for thermal image processing & irrigation management	Close-range imaging of leaves	Static close-range imagery; no continuous video or quantitative discharge data
Al-Naji et al. [3]	Soil monitoring (various plots)	Soil images under different moisture and illumination	High-resolution soil imaging	200 labeled soil images	Various illumination & soil conditions	Focused on soil-moisture estimation rather than sprinkler operation
Wang et al. [7]	Greenhouse potatoes	Thermal canopy images + environmental data	LoRa-based IoT sensors	Water stress monitoring, cloud-based	Controlled greenhouse, water stress monitoring	Greenhouse-based; no sprinkler-operation video or discharge data
Chakraborty et al. [4]	Maize fields (center pivot irrigation, LESA & MESA)	UAV multispectral + thermal images	Low-altitude UAVs	Irrigation regime impacts on crop growth	Field-scale imaging under different irrigation regimes	No continuous sprinkler-operation video or corresponding discharge measurement
OpenET [6]	Broad regions, USA	Satellite imagery (ET estimation)	MODIS, Landsat, etc.	Evapotranspiration estimation, large-scale coverage	Satellite-based temporal imagery	Satellite-based evapotranspiration estimates; no field-level sprinkler video or discharge measurements
Moscovini et al. [10]	General crops (NDVI)	Calibrated RGB images and predicted NDVI maps	Open-source ML software	Pixel-level NDVI prediction from RGB images	Calibrated RGB image acquisition under crop-monitoring conditions	No sprinkler-operation video or quantitative discharge data

Among the reviewed studies, Albuquerque et al. [2] is the most directly related to visual sprinkler-spray analysis; however, its data were obtained from a simulated irrigation setup and did not include corresponding quantitative discharge measurements. Existing datasets have advanced agricultural imaging and water-stress analysis, but they rarely combine continuous overhead video of real sprinkler operation with corresponding quantitative water-discharge measurements and reference spray-boundary information. IrriDrone was developed to address this gap and to support the evaluation of methods for spray-boundary identification, sprinkler coverage assessment, discharge estimation, anomaly detection, and temporal analysis under real agricultural conditions.

## 3 Data Description

The dataset is publicly available on Zenodo [11] as 18 ZIP archives, together with a consolidated metadata.csv file and a README.txt file. The video archives are grouped by farm, crop, active-sprinkler configuration, and annotation status. For the carrot field (farm1) and potato field (farm2), separate annotated and unannotated archives are provided for the 3-, 6-, and 9-active-sprinkler configurations. For the alfalfa field (farm3), only the 9-active-sprinkler configuration was recorded and released; no 3- or 6-active-sprinkler recordings are available for this farm. The alfalfa data are divided into three annotated archive parts and three corresponding unannotated archive parts. These archive parts are storage partitions only and do not represent predefined training, validation, or test subsets.

Each boundary-overlaid video has a corresponding standardized video without the circular spray-boundary overlay in the matching unannotated archive. The two versions contain the same underlying visual content and must be treated as paired representations of the same recording rather than as independent observations. The boundary information is embedded directly in the annotated video and is not supplied as an independent frame-level mask, polygon, coordinate file, or other machine-readable annotation format.

The dataset contains 674 paired one-rotation recordings, each provided in a boundary-overlaid and an unannotated version, resulting in 1348 publicly released MP4 files. Each released recording represents one complete sprinkler rotation extracted from an original recording containing at least two complete rotations. The starting and ending angular positions of the sprinkler vary across the released recordings; therefore, they do not share a fixed starting orientation. Because each released video contains a complete rotation, users may retain the full temporal sequence or divide it into shorter windows according to the requirements of their experiments. For example, short segments such as 15 s can be generated to investigate whether spray extent or water discharge can be estimated from limited temporal observations. These shorter segments are not provided as predefined independent samples and must be generated by users from the released videos. When generating shorter segments, all segments derived from the same released video should remain within the same training, validation, or test subset to reduce temporal leakage.

The original recordings were acquired at 4 K resolution and 120 fps. To reduce storage and download requirements, one complete rotation was extracted from each selected recording and standardized to Full HD resolution (1920 × 1080 pixels) at 50 fps for public release. The original 4K/120-fps recordings are retained by the authors but are not included in the public dataset. The released videos are distributed in 18 ZIP archives. Users can download each archive separately according to the required farm, crop, sprinkler configuration, and annotation status, or retrieve the complete Zenodo record. The accompanying metadata.csv and README.txt files can also be downloaded separately. This structure allows users to obtain only the data required for their intended experiments.

The dataset includes a consolidated metadata.csv file containing one row for each of the 674 paired one-rotation recordings. The metadata fields are Video_Name_mp4, Crop, Active_Sprinklers, Pressure, Mean_Discharge_lps, Altitude_m, Date, Time, Time_Period, Duration_s, Resolution, FPS, Latitude, Longitude, Annotated, and Status. The boundary-overlaid and unannotated versions share the same video identifier and are stored in the corresponding annotated and unannotated ZIP archives.

**Fig. 1:**
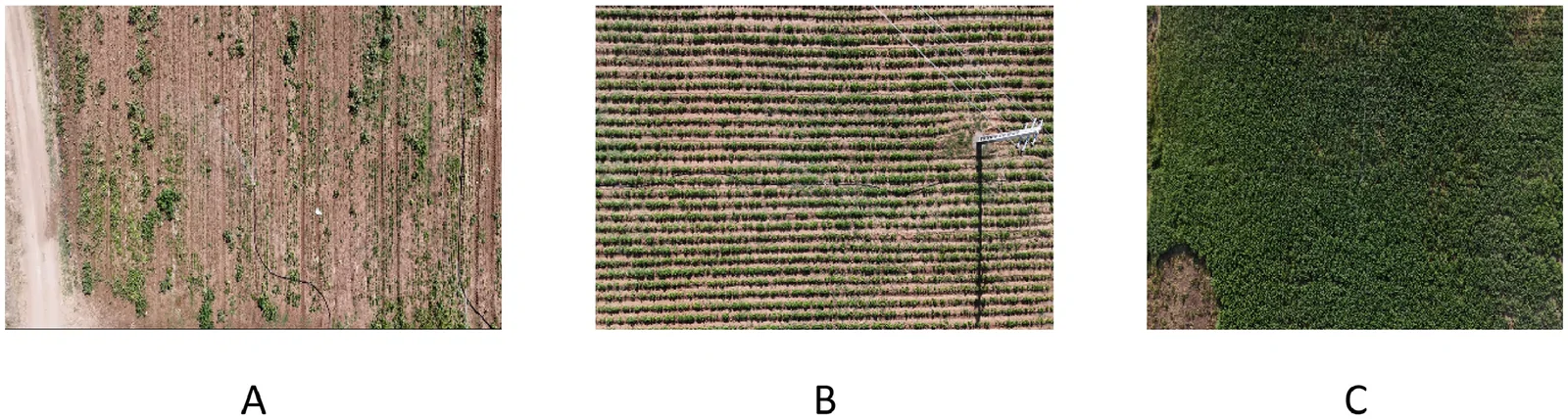
Filmed fields. A) Carrot, B) Potato, and C) Alfalfa.

**Fig. 2:**
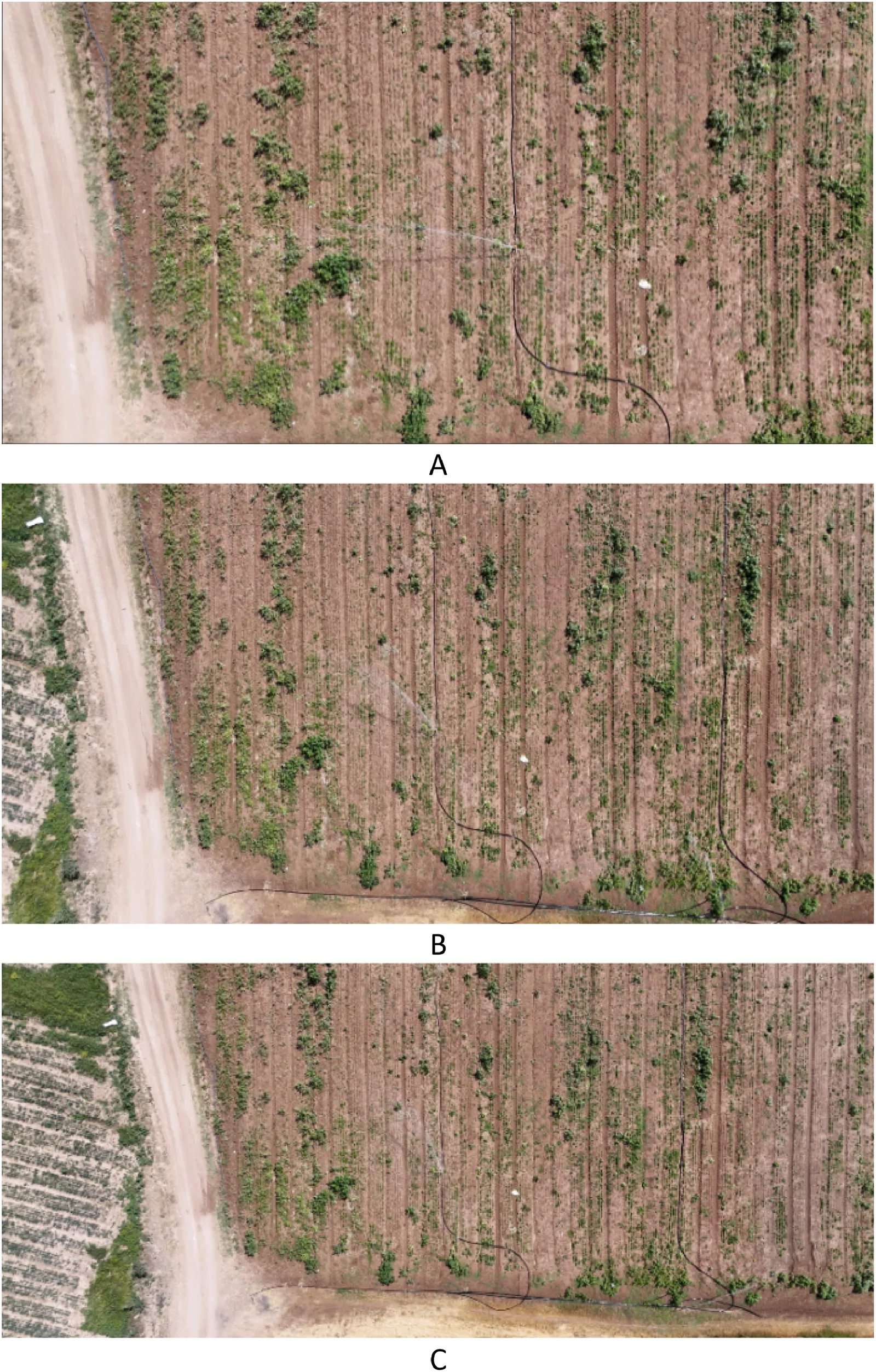
Representative frames of the carrot field recorded above a sprinkler at flight altitudes of A) 35 m, B) 45 m, and C) 55 m.

**Fig. 3:**
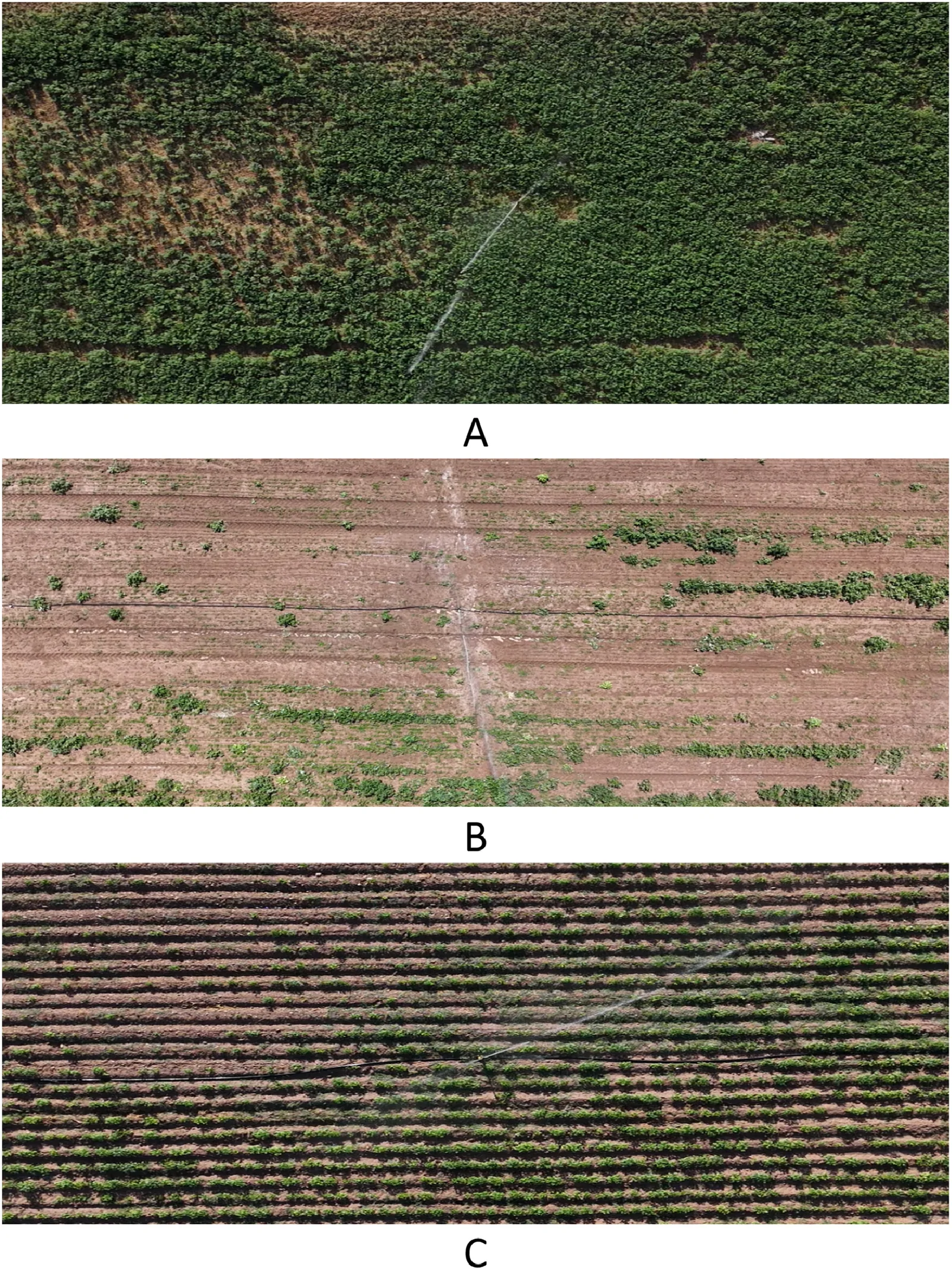
Sample images recorded at different time intervals: A) morning, B) noon, and C) evening.

**Fig. 4:**
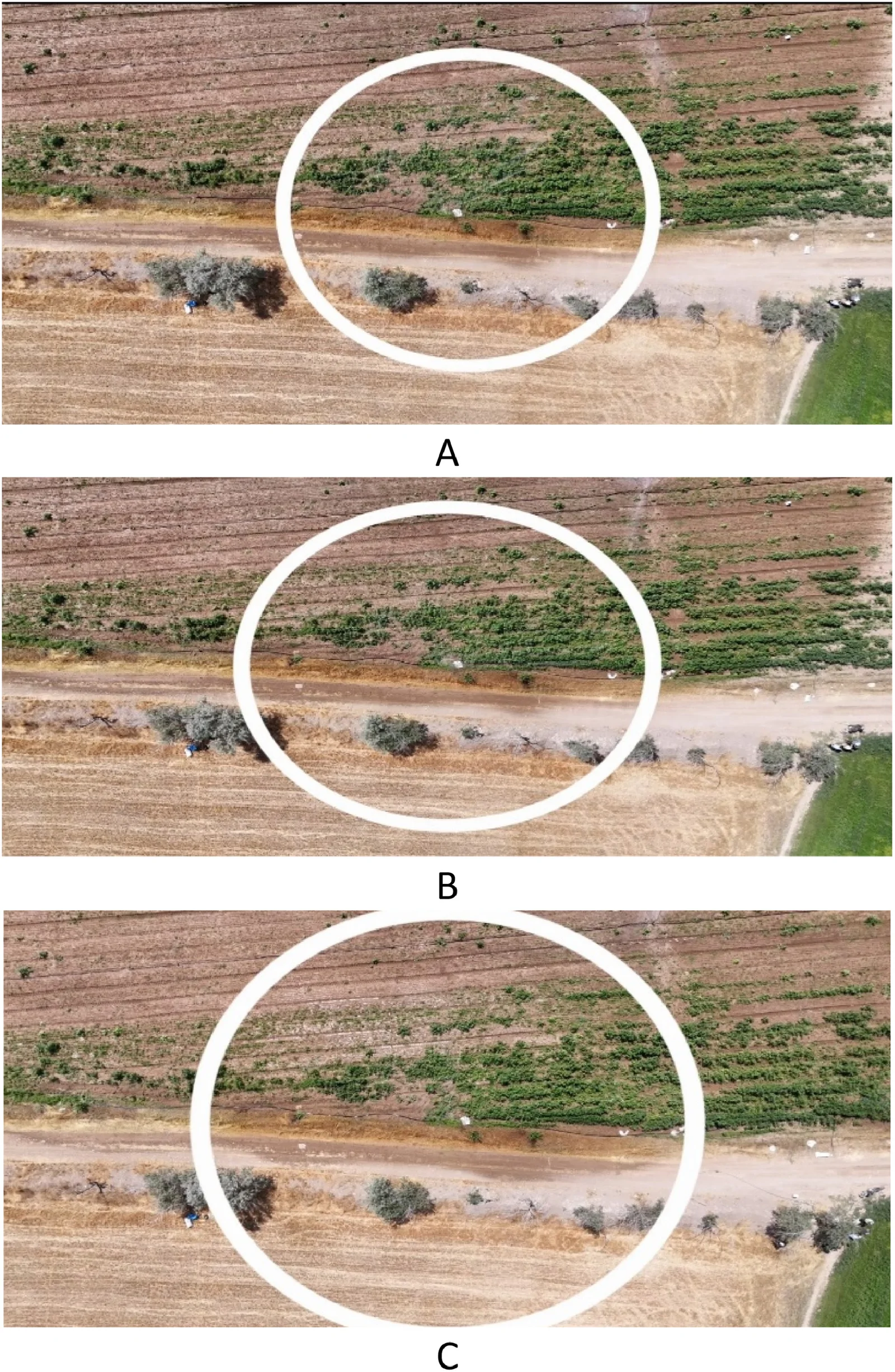
Visible sprinkler spray extent at a flight altitude of 35 m under configurations of A) 3, B) 6, and C) 9 simultaneously active sprinklers.

A source-recording identifier preserves the relationship between each released one-rotation recording and its original acquisition recording. The accompanying README.txt file describes the archive organization, filename conventions, metadata fields, measurement units, permitted values, paired-video structure, recommended use, and known limitations. Video filenames follow the pattern [farm_id]-[active_sprinklers]([original_video_id]) [video_number].mp4. For example, 2-3(1) 4.mp4 denotes released video 4 from farm 2, recorded with three simultaneously active sprinklers and extracted from original source video 1. A statistical summary of the dataset is provided in Table 2, and representative frames illustrating different acquisition conditions are shown in Fig. 1, Fig. 2, Fig. 3, Fig. 4.

**Table 2:** Statistical summary of the IrriDrone dataset by crop field and active-sprinkler configuration.

Crop	Active sprinklers	Operational pressure	No. of paired recordings	Total duration	Duration range	Altitudes	Recording-time range	Discharge range
Carrot	3	High	39	00:23:36.32	31.96–41.06 s	35, 45, 55 m	11:21 AM–11:30 AM	2.17–2.90 l/s
Carrot	6	Intermediate	90	00:51:14.80	16.06–51.40 s	35, 45, 55 m	10:59 AM–11:18 AM	2.20–2.79 l/s
Carrot	9	Low	115	00:59:07.47	12.84–81.28 s	35, 45, 55 m	11:50 AM–12:18 PM	1.71–2.77 l/s
Potato	3	High	82	00:43:03.58	25.26–37.86 s	35, 45, 55 m	8:14 AM–8:20 AM	3.28–3.33 l/s
Potato	6	Intermediate	48	00:23:41.36	24.14–34.08 s	35, 45, 55 m	8:48 AM–8:56 AM	3.11–4.09 l/s
Potato	9	Low	56	00:23:22.61	12.54–32.28 s	35, 45, 55 m	9:27 AM–9:46 AM	1.85–2.46 l/s
Alfalfa	9	Low	244	01:42:41.04	13.44–67.58 s	35, 45, 55 m	4:20 PM–4:47 PM	1.53–2.00 l/s
Total	-	-	674	05:26:47.18	12.54–81.28 s	35, 45, 55 m	8:14 AM-4:47 PM	1.53–4.09 l/s

Each paired recording is provided in two versions: one boundary-overlaid video and one corresponding unannotated video. Therefore, the 674 paired recordings comprise 1348 released MP4 files. Total duration refers to the unique video content and does not count the paired version twice.

## 4 Experimental Design, Materials and Methods

### 4.1 Study area and field selection

Data were collected from three commercially operated farms located in Ghoruchay village, Dehgolan County, Kurdistan Province, Iran. As shown in Fig. 1, the selected farms contained sprinkler-irrigated carrot, potato, and alfalfa fields. The Dehgolan Plain has a dry, cool to semi-arid climate [12]. Previous regional studies have reported a mean annual temperature of approximately 9.1–9.4 °C and mean annual precipitation of approximately 319 mm [13]. These long-term regional values characterize the broader study area and do not represent on-site meteorological measurements during the individual drone flights. The three crop fields provided differences in canopy structure and field-surface appearance.

Recordings were collected during one agricultural season on the dates reported in metadata.csv. Acquisition was not necessarily completed within a single day for every field; in some cases, recording continued on another day because of UAV battery limitations. Recordings were obtained at different times of day and under the sprinkler configurations available for each field. The specific crop cultivars were not recorded during data acquisition and are therefore unavailable.

The irrigation system was operated with 3, 6, or 9 simultaneously active sprinklers, as illustrated in Fig. 4. The pump setting was kept unchanged while the number of active sprinklers was varied to establish three relative operational pressure conditions. With three active sprinklers, the pressure per sprinkler was highest and the visible spray radius was largest. The six-sprinkler configuration represented the intermediate condition, whereas the nine-sprinkler configuration produced the lowest pressure per sprinkler and the smallest visible spray radius. These categories describe relative operational pressure conditions; pressure was not directly measured in bar or kPa. Quantitative discharge was measured separately in liters per second as described in Section C.

### 4.2 Aerial video acquisition

A DJI Air 3S drone equipped with a wide-angle camera was used to acquire the aerial videos at a native resolution of 4 K and a frame rate of 120 fps. Camera exposure settings were controlled automatically by the drone and were not retained as part of the released metadata. As shown in Fig. 2, video acquisition was conducted at nominal flight altitudes of 35, 45, and 55 m. During each recording, the drone hovered above the target sprinkler with the camera directed vertically downward. Lower altitudes were avoided to reduce the risk of direct water-spray exposure and insufficient field coverage, while higher altitudes were avoided because of local operational and flight-safety constraints.

Each original recording captured at least two complete rotations of the target sprinkler. Drone metadata, including flight altitude, GPS coordinates, and recording time, were retained and associated with the corresponding recording through its unique identifier. Recordings were obtained during morning, noon, and evening periods, as illustrated in Fig. 3, to represent variations in illumination, shadow length, and surface reflection. The clock time associated with each released recording is provided in metadata.csv. The extraction and standardization of the publicly released one-rotation videos are described in Section D.

### 4.3 Discharge measurement and temporal association

Water discharge associated with each sprinkler recording was determined using a 20-L volumetric method consistent with the approach described by Shahrokhnia et al. [14]. Immediately after each 3–5-min original video recording, and without any intentional delay or change in the pump setting or the number of active sprinklers, the time required to fill a 20-L container was measured in three consecutive trials. The mean filling time was calculated as:

(1) $$\bar{t}=\left(t_{1}+t_{2}+t_{3}\right)/3$$

where $t_{1}$, $t_{2}$, and $t_{3}$ are the three measured filling times in seconds, and $\bar{t}$ is their mean. The corresponding water discharge was calculated as:

(2) $$Q=V/\;\bar{t}$$

where V equals to 20 liters as the container volume and Q is the estimated discharge in liters per second (L/s). The three filling-time measurements were closely clustered, with differences generally limited to hundredths of a second. The resulting discharge value was associated with the corresponding original recording through its unique source-recording identifier and assigned to the released one-rotation video derived from that recording. No inline flowmeter was used. Because discharge was measured immediately after rather than continuously during video acquisition, the reported values represent temporally associated, configuration-matched measurements rather than frame-level synchronized data.

### 4.4 Video preparation and spray-boundary overlay

After acquisition, the original videos underwent quality screening, one-rotation extraction, standardization, spray-boundary overlay, review, and metadata association, as described below.

1. **Quality screening and one-rotation extraction**: The original recordings were reviewed before inclusion in the dataset, and severely corrupted or unusable recordings were excluded. From each selected original recording containing at least two complete sprinkler rotations, one complete rotation was extracted for public release. The starting and ending angular positions were not fixed across the released recordings.
2. **Video standardization and paired-video preparation**: Video extraction and standardization were performed using VideoPad Video Editor (NCH Software). Each extracted one-rotation sequence was exported in MP4 format at Full HD resolution (1920 × 1080 pixels) and 50 fps. The standardized video was retained as the unannotated version and subsequently used to produce the corresponding boundary-overlaid version. The two files contain the same underlying temporal sequence and constitute a paired recording.
3. **Spray-boundary overlay**: One member of the research team created the initial spray-boundary overlay on each standardized video using the S Pen and drawing functionality of the built-in video player on a Samsung Galaxy Tab A tablet (model SM-P205). The boundary was represented by a white circle and was determined using the visible wetted area, observed spray extent, and field observations made during data acquisition. The circular overlay was applied at the video level and remained visible throughout all frames of the exported boundary-overlaid video; it was not independently redrawn for each frame. No separate mask, polygon, coordinate file, or other machine-readable annotation file was generated. The name and version of the built-in video-player application were not retained.
4. **Boundary review and consensus**: Two additional members of the research team reviewed each boundary-overlaid video. When a reviewer identified a discrepancy, the video was returned to the original annotator for correction. The revised boundary was accepted after consensus among the three team members. No substantial disagreements were encountered during this process. Because the reviewers assessed and refined a common initial boundary rather than independently producing separate annotations, no formal inter-annotator agreement metric, such as Intersection over Union (IoU), was calculated.
5. **Metadata association**: Flight altitude, GPS coordinates, recording clock time, active-sprinkler configuration, operational pressure category, and mean water-discharge value were associated with each paired one-rotation recording through a unique identifier. These associations are explicitly provided in metadata.csv. The discharge value represents the volumetric measurement obtained immediately after the corresponding original recording under unchanged irrigation-system settings, as described in Section C.

## Limitations

Several limitations should be considered when using this dataset. All recordings were acquired from three crop fields in a single geographical region during one agricultural season. Recordings were collected on the dates reported in metadata.csv, and in some cases acquisition for a field continued on another day because of UAV battery limitations. Although the dataset includes multiple recording dates, it does not capture seasonal variability or repeated observations across different crop-growth stages. Although the three fields provide differences in crop canopy and field appearance, the dataset does not capture broader regional variation in crop cultivars, field conditions, climate, or irrigation practices. Comparisons among the three crops may also reflect differences among their respective fields and recording dates. Models developed using IrriDrone should therefore be externally validated before application to other regions, seasons, or cropping conditions.

Second, aerial video acquisition was intentionally restricted to a standardized overhead geometry in which the drone hovered directly above the target riser and the camera was directed vertically downward. This configuration is consistent with the intended use of IrriDrone for overhead assessment of sprinkler spray extent and discharge. Consequently, the dataset does not represent oblique viewing angles or alternative sensing modalities such as thermal or multispectral imagery. Recordings were obtained under visually calm conditions without observable wind-induced disruption to normal sprinkler operation; therefore, the dataset is not intended to characterize spray patterns under strong or highly variable wind conditions.

Third, the publicly released videos were standardized to Full HD resolution (1920 × 1080 pixels) at 50 fps and contain one complete sprinkler rotation. The original 4K/120-fps recordings are not included in the public release. Consequently, the released videos may not preserve all the spatial and temporal detail available in the original acquisitions, which may limit analyses requiring fine-scale characterization of spray droplets or rapid spray dynamics.

Fourth, the circular spray-boundary overlays were initially created by one member of the research team and subsequently reviewed by two additional team members until consensus was reached. Because the reviewers assessed and refined a common initial boundary rather than independently producing separate annotations, no formal inter-annotator agreement metric, such as Intersection over Union (IoU), was calculated. Moreover, the final boundary is embedded directly in the boundary-overlaid video and is not provided as an independent frame-level mask, polygon, or machine-readable annotation file. This format may require additional processing by users intending to perform conventional pixel-level segmentation or quantitative annotation analysis. Future dataset extensions could include independent duplicate annotations, formal agreement metrics, and machine-readable masks.

Finally, the dataset contains 674 paired one-rotation recordings from three crop fields and three active-sprinkler configurations. The 3, 6, and 9 sprinkler-configurations represent the relative highest, intermediate, and lowest operational pressure conditions per sprinkler, respectively; however, pressure was not directly measured in bar or kPa. Although corresponding discharge values were obtained using repeated volumetric measurements, these three configurations do not represent the full range of sprinkler types, pump settings, pressure conditions, or discharge rates that may be encountered in commercial irrigation systems.

## Ethics Statement

This work did not involve human subjects, animal experiments, or data collected from social media platforms. All agricultural fields included in the study were commercially operated farms, and data collection was conducted with permission from the respective landholders. Drone flights were conducted in accordance with applicable local airspace regulations and safety guidelines. The authors have read and complied with the ethical requirements for publication in Data in Brief.

## CRediT Author Statement

**Somayeh Afshar:** Conceptualization, Data curation, Formal analysis, Methodology, Validation, Visualization, Writing original draft, Review, and Editing, Resources, Software; **Mohsen Ramezani:** Supervision, Validation, Conceptualization, Data curation, Formal analysis, Methodology, Writing original draft, Review, and Editing; **Kaveh Mollazade:** Supervision, Validation, Conceptualization, Formal analysis, Writing, Review, and Editing; **Atefe Moradiani:** Methodology, Formal analysis.

## Data Availability

ZenodoIrriDrone: Overhead Drone-Based Video Dataset for Precision Sprinkler Irrigation Assessment (Original data).

## References

[1] Kamyshova G. (2022). Artificial neural networks and computer vision's-based phytoindication systems for variable rate irrigation improving. IEEE Access.

[2] Albuquerque C.K.G. (2020). 2020 IEEE International Workshop on Metrology for Agriculture and Forestry (MetroAgriFor).

[3] Al-Naji A. (2021). Soil color analysis based on a RGB camera and an artificial neural network towards smart irrigation: a pilot study. Heliyon.

[4] Chakraborty M., Khot L.R., Peters R.T. (2020). Assessing suitability of modified center pivot irrigation systems in corn production using low altitude aerial imaging techniques. Inf. Process. Agric..

[5] Kumar M.S. (2024). Visualization and characterization of agricultural sprays using machine learning based digital inline holography. Comput. Electron. Agric..

[6] Melton F.S. (2022). OpenET: filling a critical data gap in water management for the western United States. JAWRA J. Am. Water Resour. Assoc..

[7] Wang L. (2023). Extraction of 3D distribution of potato plant CWSI based on thermal infrared image and binocular stereovision system. Front. Plant Sci..

[8] Ali Z.A. (2024). AI-based UAV swarms for monitoring and disease identification of brassica plants using machine learning: a review. Comput. Syst. Sci. Eng..

[9] Cucho-Padin G. (2020). Development of an open-source thermal image processing software for improving irrigation management in potato crops (*Solanum tuberosum* L.). Sensors.

[10] Moscovini L. (2024). An open-source machine-learning application for predicting pixel-to-pixel NDVI regression from RGB calibrated images. Comput. Electron. Agric..

[11] S. Afshar, M. Ramezani, K. Mollazade, A. Moradiani, IrriDrone: overhead drone-based video dataset for precision sprinkler irrigation assessment, version 1.0, Zenodo, 2026. 10.5281/zenodo.22017449

[12] Amini A., Homayounfar V. (2017). The groundwater balance in alluvial plain aquifer at Dehgolan, Kurdistan, Iran. Appl. Water Sci..

[13] Naserabadi F., Ghazavi R., Zakerinia M. (2025). Assessment of climate change impact on groundwater quantitative status in the Dehgolan aquifer using the MODFLOW model. J. Watershed Manag. Res..

[14] Shahrokhnia M.A., Akbari M., Abbasi F. (2023). Measuring the volume of irrigation water and water productivity of onion farms in three regions of Fars province. Iran. Water Res. J..

